# Tapered Cuff versus Conventional Cuff for Ventilator-Associated Pneumonia in Ventilated Patients: A Meta-Analysis of Randomized Controlled Trials

**DOI:** 10.1155/2019/7876417

**Published:** 2019-01-22

**Authors:** Wei Min Huang, Xu An Huang, Yan Ping Du, Liu Xia Li, Fang Fang Wu, Shao Qing Hong, Fang Xuan Tang, Zhang Qiang Ye

**Affiliations:** ^1^Department of Hospital Infection-Control, Xiamen Haicang Hospital, Xiamen, China; ^2^Medical College of Xiamen University, Xiamen, China; ^3^Department of Respiratory Medicine, Zhongshan Hospital, Xiamen University, Teaching Hospital of Fujian Medical University, Xiamen, China; ^4^Department of Respiratory Medicine, Fujian Medical University Union Hospital, Zhongshan Hospital Xiamen University, Xiamen, China

## Abstract

**Background:**

Microaspiration of secretions around the tracheal cuff is a multifactorial process. Tracheal cuff shape might take a major part in its occurrence. The rationale for producing a taper-shaped cuff is established on the assumption that compared to a conventional cuff with a single fixed diameter, a continuum of minimum-to-maximum diameter sections might better fit the tracheal walls.

**Objectives:**

The primary objective of this meta-analysis was to compare ventilator-associated pneumonia (VAP) between tapered-cuff intubation and conventional-cuff intubation. The secondary objective was to compare intensive care unit (ICU) mortality between tapered-cuff intubation and conventional-cuff intubation.

**Methods:**

We searched the Cochrane Library, Embase, MEDLINE database through the PubMed search engine, and CINAHL from inception to April 2018. Randomized trials comparing VAP and ICU mortality between tapered-cuff intubation and conventional-cuff intubation in intubated adults were included. Two review authors assessed study quality and abstracted databasing on prespecified criteria independently.

**Results:**

We pooled summary estimates from 5 trials evaluating tapered-cuff involving 774 participants. Compared to VAP, no statistically significant difference was observed between the tapered-cuff and conventional-cuff groups (OR 0.82, CI 0.61–1.12, *z* = 1.24, and *p*=0.21). No statistically significant difference was observed between the tapered-cuff and conventional-cuff groups with ICU mortality (OR 0.77, CI 0.55–1.08, *z* = 1.49, and *p*=0.14).

**Conclusions:**

In this meta-analysis, the tapered-cuff tracheal tube may not be superior to the standard-cuff tracheal tube in reducing VAP and ICU mortality.

## 1. Introduction

Ventilator-associated pneumonia (VAP) occurs after starting mechanical ventilation. Prevalence of VAP and its mortality was 11% and 78.9%, respectively [1]. Different risk factors lead to VAP. The specific risk factors are associated with their underlying diseases and invasive medical procedures, which they undergo [2]. Interventions such as selective decontamination of subglottic space, continuous or intermittent suction of subglottic space [3], and synchronized mucus aspiration in the distal end of an endotracheal tube lead to decreasing VAP [4].

The primary mechanism of VAP pathogenesis is colonized oropharyngeal secretion aspiration [5]. Studies undertaken with the goal of preventing VAP have been focused on improving tracheal sealing through controlling cuff pressure or modifying the cuff shape [6, 7]. Although microaspiration of secretions around the tracheal cuff is a multifactorial process, tracheal cuff shape might take a major part in its occurrence [8]. An in vitro study suggests a beneficial effect of a tapered-cuff tube in reducing subglottic fluid leakage by providing a permanent sealing zone between the cuff and the tracheal wall [9]. The previous study suggested that the short-term use of taper-shaped polyvinyl chloride cuffs in surgical patients resulted in more effective sealing of the tracheal lumen in comparison with traditional barrel-shaped polyvinyl chloride cuffs [10]. However, other animal and in vitro studies found that further evaluation is needed to determine whether a reduction in VAP can be demonstrated when taper-shaped cuffs are used because studies have yielded conflicting results [8, 11].

## 2. Methods

### 2.1. Electronic Search

We carried out a literature search using the Cochrane Library, Embase, MEDLINE database through the PubMed search engine, and CINAHL from inception to April 2018. No restrictions were placed on the language of the publications. The following medical subject headings (MeSH) were searched: randomized controlled trial, tapered cuff, conical cuff, cylindrical cuff, spherical-shaped cuff, conventional cuff, standard cuff, and ventilator-associated pneumonia. Since our study was a meta-analysis, the approval of the ethics committee was not available.

### 2.2. Inclusion Criteria/Exclusion Criteria

We included randomized controlled trials (RCTs) that involved the use of a tapered cuff and a conventional cuff for intubation. Articles were included if they were in English and were full-text articles reporting original research. We considered for inclusion of all adults. If the primary outcome measures were not VAP, then these studies were excluded. Editorials, letters, and retrospective studies were excluded. Any disagreement about whether the trials meet the inclusion or exclusion criteria between the two reviews was resolved by discussing with a third reviewer. We resolved the problem through the agreement by two reviewers.

### 2.3. Data Extraction

Two reviewers independently evaluated the included studies and extracted data into RevMan (Review Manager: Cochran handbook for systematic reviews). Any disagreement was resolved by discussion with a third reviewer. If further data were required, communication through e-mail would be carried out with the authors. The following items were extracted from the tapered-cuff group and the conventional-cuff group: first author, publication year, country, study design, kind of intensive care unit (ICU), sample size, endotracheal tube type, internal diameter of the tracheal tube, cuff pressure, PEEP, VAP assessment, and duration of follow-up.

### 2.4. Outcomes' Measure

The primary endpoint of this meta-analysis VAP was defined based on clinical, radiological, and laboratory findings. The secondary outcomes were ICU mortality.

### 2.5. Quality Assessment

All studies were assessed with the Jadad scale scoring system [12] in which the best study quality scored was 5 points. Studies with a score ≥3 points were considered as high-quality research and were enrolled. Studies were also classified by two authors as having a low risk of bias, an unclear risk of bias, or a high risk of bias based on the Cochrane tool. The Cochrane tool takes into account random sequence generation, concealment of the allocation sequence, blinding of participants and personnel, blinding of outcome assessment, incomplete outcome, and selective reporting.

### 2.6. Statistical Analysis

For each included study, odds ratio (OR) and 95% confidence interval (CI) were calculated for dichotomous outcomes. Statistical heterogeneity was assessed using the *I*^2^ value. When *I*^2^ value ≤ 50%, the included studies were considered to have no statistical heterogeneity and used the fixed-effect model to estimate the overall summary effect sizes. Otherwise, a random-effect model has used a subgroup analysis or sensitivity analysis. The risk of a publication bias was assessed by visual inspection of funnel plots of effect size. We used Review Manager software (RevMan 5.3), and *p* value <0.01 was considered significant.

## 3. Results

### 3.1. The Result of the Search

**Figure 1:**
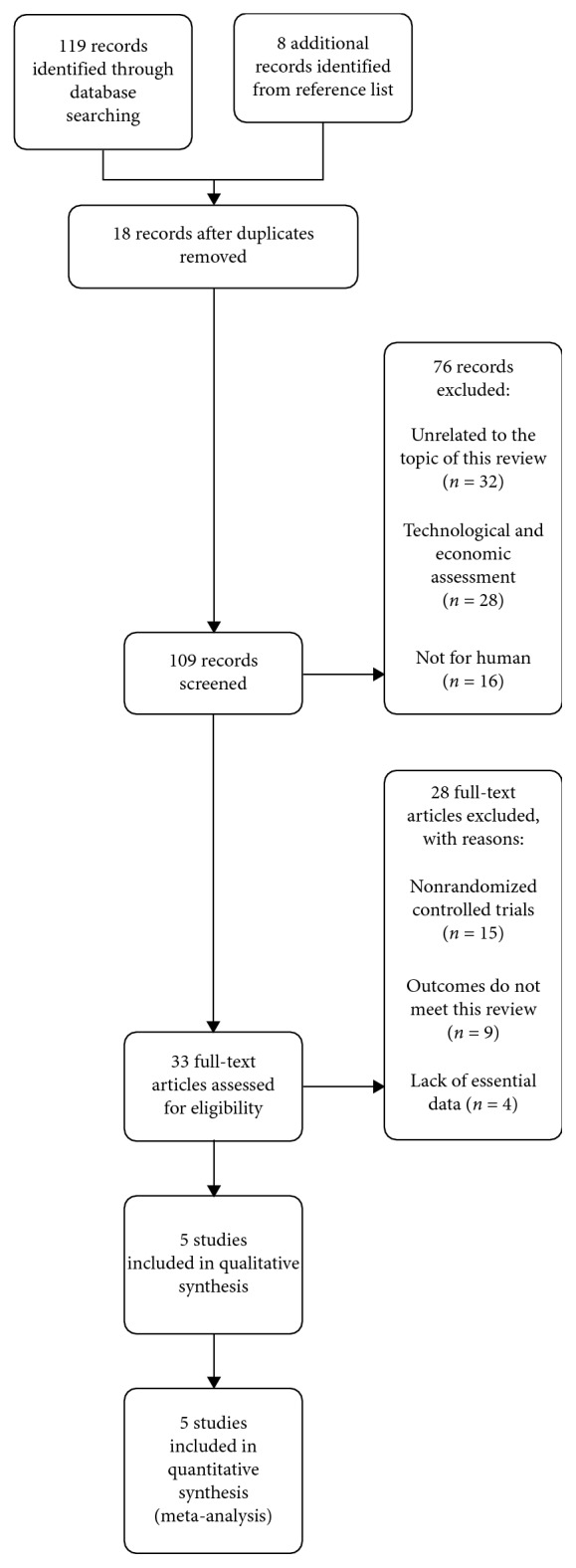
The graph showing a flow diagram of detailed search and exclusion criteria.

A total of 127 potentially related articles were preliminarily screened on the search of the database. Eighteen articles were excluded for a duplicate. After reading the abstracts, 76 articles were excluded, whereas 33 articles were excluded for full-text scrutiny. Finally, the search strategy identified that a total of 5 published RCTs were included in the final analysis [13–17]. The details of the search and exclusion criteria are displayed in the flow diagram (Figure 1).

### 3.2. Characteristics of Selected Studies

We identified 5 RCTs. All selected studies in our meta-analysis were published from 2013 to 2017. The follow-up period ranged from 1 week to 2 months after using ventilation. The selected study characteristics are summarized in Table 1.

### 3.3. Quantitative Data Synthesis

**Figure 2:**
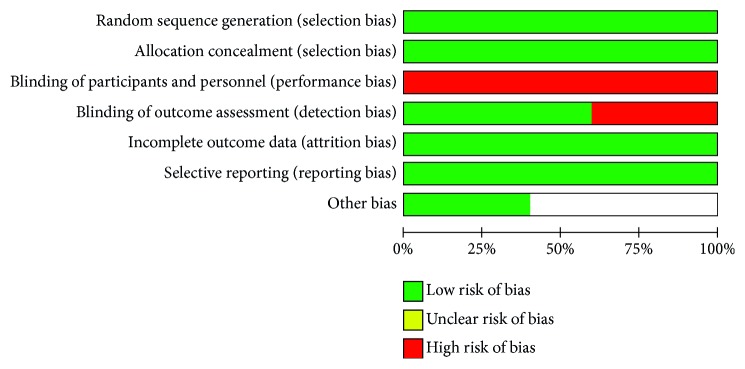
The graph showing a risk of bias graph.

**Figure 3:**
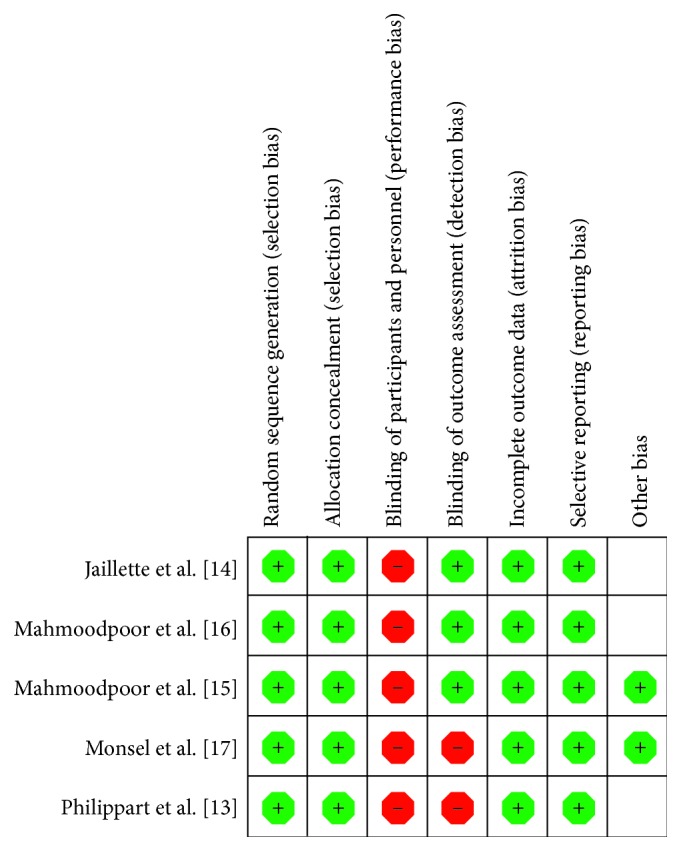
The graph showing a risk of bias summary.

The detailed risk of bias abuts the methodological quality of the included studies that are elaborated and summarized, respectively, in Figures 2 and 3.

### 3.4. Meta-Analysis Results

#### 3.4.1. The Primary Endpoint

**Figure 4:**
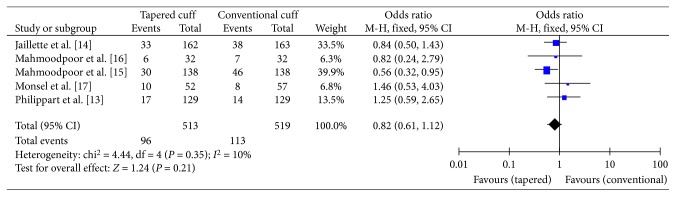
The graph showing a forest plot of relative risk with confidence interval for VAP.

The primary endpoint “VAP” was reported in all five studies. A total of 384 patients in the tapered-cuff group and 390 patients in the conventional-cuff group were available to compare VAP. No statistically significant difference was observed between the tapered-cuff and conventional-cuff groups (OR 0.82, CI 0.61–1.12, *z* = 1.24, and *p*=0.21) (Figure 4).

#### 3.4.2. The Secondary Endpoint

**Figure 5:**
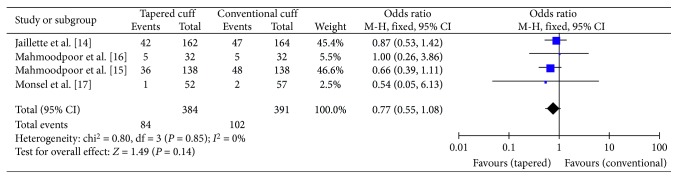
The graph showing a forest plot of relative risk with confidence interval for ICU mortality.

The secondary endpoint “ICU mortality” was reported in all four studies. A total of 384 patients in the tapered-cuff group and 391 patients in the conventional-cuff group were available to compare ICU mortality. No statistically significant difference was observed between the tapered-cuff and conventional-cuff groups (OR 0.77, CI 0.55–1.08, *z* = 1.49, and *p*=0.14) (Figure 5).

## 4. Discussion

Our study found that tapered-cuff tracheal tube compared with a conventional-cuff tracheal tube did not reduce the VAP rate and ICU mortality.

We conducted this meta-analysis because the tracheal cuff is the crucial interface between the tube and the tracheal wall that may be responsible for the leakage of contaminated secretions, which lead to colonization and possibly to VAP. New cuffs were developed recently to reduce microaspiration of secretions around the cuff of the endotracheal tube. A tapered shape theoretically ensures that the cuff and trachea share the same diameter, thereby leading to smaller folds and improving tracheal sealing [18].

In our meta-analysis, we found that tapered-cuff tracheal tubes, compared with conventional-cuff tracheal tubes, did not reduce VAP. But an in vitro study and a clinical study found that using tapered-cuff tracheal tubes decreased leakage around the tracheal cuff compared with the conventional cuff [9, 10, 19]. One potential explanation for the absence of the beneficial effect of tapered cuff is the reduced contact zone between the tracheal wall and the cuff, which might lead to increased mobility of tapered cuff in comparison with the standard cuff. Movement of the tracheal cuff has been found to result in potentially harmful cuff pressures and can be a risk factor for microaspiration and leakage [20, 21]. Indeed, the clinical trial was achieved in patients heavily anesthetized for surgery, and the tracheal cuffs were systematically fixed in in vitro studies, so the mobility of the tracheal tube was very limited [9, 10, 19].

The rationale for producing a taper-shaped cuff was established on the assumption that compared to a conventional cuff with a single fixed diameter, a continuum of minimum-to-maximum diameter sections might better fit the tracheal walls. However, compared with other spherical- or cylindrical-shaped cuffs, tapered cuffs had the lowest tracheal wall contact area [22]. This small contact area might result in cuff-pressure fluctuation and cuff slippage. These effects are possibly detrimental, so continuous cuff-pressure control and close monitoring would be required when tapered-cuff tubes were to be used.

Different risk factors lead to ICU mortality. The specific risk factors are associated with their underlying diseases and VAP. We know that VAP is the most important risk factor for ICU mortality. In our meta-analysis, there was no difference in VAP between the tapered-cuff group and the conventional-cuff group. We estimate that the microaspiration between the two groups has no difference. So the ICU mortality between the two groups has no difference.

The blinding of participants and personnel was high risk because blinding of ICU physicians was not feasible during intubation. However, we estimate this bias to be minor, as the primary endpoint VAP and the secondary endpoint ICU mortality were blindly evaluated by physicians.

### 4.1. Limitations

The current meta-analysis has some limitations. First, some included studies used polyurethane cuffs while others used polyvinyl chloride cuffs. Although bench studies suggested that polyurethane tapered cuffs had no benefits for microaspiration reduction, whether the cuff material might impact tracheal sealing remains to be investigated further. Second, in the conventional groups, one included study used spherical cuffs while others used cylindrical cuffs. Given that spherical and cylindrical cuffs are used worldwide, with proven good performances, have been compared in multiple trials [23], we considered that such control group management would not introduce bias into our meta-analysis.

## 5. Conclusion

The tapered-cuff tracheal tube may not be superior to the standard-cuff tracheal tube in reducing VAP and ICU mortality.

## Figures and Tables

**Table 1:** Characteristics of the five randomized controlled trials included in the meta-analysis.

Author, year	Design	Country	Kind of ICU	No. of patients	Endotracheal tube type	Internal diameter of tracheal tube	Cuff pressure	PEEP	VAP assessment	Duration of follow-up	Quality assessment
Jaillette et al. 2017 [14]	Multicenter, randomized, cross-over, open-label	France	Mixed ICU	Tapered (*n*=162), standard (*n*=164).	PVC tapered-cuffs (TaperGuard; Covidien, Athlone, Ireland); PVC standard cuffs (Hi-Lo; Covidien, Athlone, Ireland)	7.5 and 8 mm	Tapered 29 (26–30) cmH_2_O, conventional 28 (25–30)cmH_2_O	>5 cmH_2_O	Using clinical, radiographic, and microbiological criteria.	Until 28 days or ICU discharge	Good

Mahmoodpoor et al., 2013, [16]	Randomized, single-blind	Iran	Mixed ICU	Tapered (*n*=32), standard (*n*=34).	TaperGuard tubes (PU, cone-/taper-shaped cuffs with subglottic suction ports); SealGuard tubes (PU, cylindrical-/barrel-shaped cuffs with subglottic secretion suction ports).	Male 8.0 to 8.5 mm, female 7.0 to 7.5 mm.	Tapered (24.07 ± 0.48 cmH_2_O), conventional (24.10 ± 0.49 cmH_2_O)	5 cmH_2_O	VAP was defined based on clinical, radiological, and laboratory findings based on CPIS.	Until ICU discharge	Good

Mahmoodpoor et al., 2017, [15]	Randomized, single-blind	Iran	Surgical ICU	Tapered (*n*=138), conventional (*n*=138).	TaperGuard tubes; conventional high-volume low-pressure endotracheal tubes.	Male 8.0 to 8.5 mm, female 7.0 to 7.5 mm.	Tapered (23.7 ± 2.3 cmH_2_O), conventional (27.0 ± 4.7 cmH_2_O)	5 cmH_2_O	Suspected by clinical infection pulmonary score (CPIS)	Until ICU discharge	Good

Monsel et al., 2016, [17]	Randomized, single-blind	France	Surgical ICU	Tapered (*n*=52), standard (*n*=57).	TaperGuard (Covidien, Ireland); standard cuffs with high contour Brandt endotracheal tubes incorporating a polyvinyl chloride spherical-shaped cuff (Mallinckrodt Medical,USA)	The endotracheal tube size was chosen according to professional guidelines.	25 cmH_2_O	Tapered 5.6 (5.0–6.3) cmH_2_O, conventional 5.0 (5.0–6.0) cmH_2_O	VAP was confirmed when Johanson criteria were associated with a significant concentration of bacteria isolated from lower respiratory tract.	Lengths of mechanical ventilation and ICU stay.	Good

Philippart et al., 2015, [13]	Randomized, multicenter, open-lablel	France and Tunisia	Medical surgical ICUs	Tapered (*n*=129), standard (*n*=129)	TaperGuard (Covidien, Ireland); cylindrical (Hi-Lo; Covidien, Dublin, Ireland)	The patients received tracheal intubation with a 7.5–8.0 mm diameter.	The cuff was inflated to a pressure of 25–30 cmH_2_O	≥5 cmH_2_O	VAP was confirmed when the quantitative culture was at least 10^4^ cfu/ml in a bronchoalveolar lavage or 10^5^ dfu/ml in a quantitative tracheal aspirate.	Until ICU discharge	Good

## Abbreviations

VAP: Ventilator-associated pneumonia
ICU: Intensive care unit
MeSH: Medical subject headings
RCTs: Randomized controlled trials
RevMan: Review Manager
OR: Odds ratio
CI: Confidence interval.
